# Which Characteristics are Associated With Going Outside for People Living With Dementia in Nursing Homes? A Cross-Sectional Study

**DOI:** 10.1177/07334648241298107

**Published:** 2024-11-12

**Authors:** Melanie van der Velde-van Buuringen, Debbie Verbeek-Oudijk, Hilde Verbeek, Wilco P. Achterberg, Monique A. A. Caljouw

**Affiliations:** 1 University Network for the Care sector Zuid-Holland, 4501 Leiden University Medical Center, Leiden, the Netherlands; 2 Department of Public Health and Primary Care, 4501 Leiden University Medical Center, Leiden, the Netherlands; 3 Zorginstellingen Pieter van Foreest, Delft, the Netherlands; 4 2877 Netherlands Institute for Social Research, the Hague, the Netherlands; 5 Department of Health Services Research, CAPHRI, Faculty of Health, Medicine and Life Sciences, Maastricht University, Maastricht, the Netherlands; 6 Living Lab in Ageing and Long-Term Care, Maastricht University, Maastricht, the Netherlands; 7 LUMC Center for medicine for Older People, 4501 Leiden University Medical Center, Leiden, the Netherlands

**Keywords:** dementia, nursing homes, quality of life, going outside

## Abstract

This cross-sectional study explores the frequency of going outside and characteristics that are associated with going outside for people living with dementia in nursing homes in the Netherlands. A subsample of a national survey in 353 nursing homes was used (*N* = 693). Two-thirds (66.5%) go outside often. Compared to those who rarely or never go outside, participants who go outside often receive visits more often (odds ratio (OR) 1.68, 95% confidence interval (CI) 1.02–2.75), have less severe physical impairments (severe vs. mild: OR 0.17, 95% CI 0.04–0.73; very severe vs. mild: OR 0.11, 95% CI 0.03–0.49), use less pain medication (OR 0.61, 95% CI 0.38–0.98), experience higher positive affect (OR 1.10, 95% CI 1.03–1.17), and feel less at home (OR 0.86, 95% CI 0.76–0.97). These findings are the first step in developing effective interventions that will contribute to people living with dementia going outside more often.

**What this paper adds**

• Two in three people living with dementia in nursing homes go outside often.
• Receiving visits, pain medication, the level of physical impairments, positive affect, and feeling at home were independently associated with the frequency of going outside.
• The level of physical impairments explained the largest proportion of variability in the frequency of going outside.

**Applications of study findings**

• Identifying characteristics that act as barriers or enablers to go outside is crucial for developing effective interventions to make going outside a normal part of daily nursing home practice for people living with dementia.
• Given that the level of physical impairments explained the largest proportion of the variability in the frequency of going outside, interventions should start with focusing on addressing this important factor.

## Introduction

There are 300,000 people living with dementia in the Netherlands, of whom 80,000 live in nursing homes (Alzheimer, 2021). These nursing homes are often mixed, housing somatic and psychogeriatric residents, and often have specialized wards for people living with dementia. Dementia is a major neurocognitive disorder, with a progressive decline in various cognitive functions influencing intellectual, social, and physical functioning (Banerjee et al., 2009; Sachdev et al., 2014). Due to this decline, performing normal daily activities, such as going outside independently, becomes increasingly challenging.

In recent years, an increasing number of studies focus on the effects of going outside on people living with dementia in nursing homes. The term “outside” can refer to various settings such as a garden, balcony, or short walks outside the grounds of the nursing home. Overall, first results appear to suggest positive effects of garden use on quality of life (QoL), behavioral and psychological symptoms of dementia (BPSD), other outcomes (stress, sleep, and mood), and physical and cognitive abilities (Nicholas et al., 2019; Velde-van Buuringen et al., 2023). For example, in one pilot study evaluating the effects of garden visits, staff reported that garden visits reduced residents’ depression, anxiety/agitation, and aggression/anger significantly more than other behavioral problems (Liao et al., 2020). Another study investigated the effects of viewing a garden on physiological stress (Goto et al., 2018). People living with dementia viewed the specially constructed garden two times a week for 15 minutes together with a caregiver and researcher (Goto et al., 2018). Garden observation with the door open relieved physiological stress, as reflected in a sustained drop in the pulse rate (Goto et al., 2018).

Several qualitative studies exploring benefits, personalization, and the effect of garden use on QoL revealed themes around experiences and possible mechanisms of the positive effects of going outside from the perspectives of people living with dementia, staff members, and relatives (Evans et al., 2019; Hendriks et al., 2016; van der Velde-van Buuringen et al., 2020). Garden use seems to have a positive effect on QoL by facilitating a sense of freedom, social interaction, a calming effect, reminiscence, and pleasure (Evans et al., 2019; Hendriks et al., 2016; van der Velde-van Buuringen et al., 2020; Velde-van Buuringen et al., 2023).

Despite these benefits, it seems that going outside is still not a normal part of daily nursing home practice. Furthermore, there is a paucity of data on the access to, and use of the outside area (van den Berg et al., 2020). Sixty percent of all residents living in a nursing home experience health-related barriers to going outside, while 34% experience barriers related to a lack of company (Verbeek-Oudijk & Koper, 2019). Also, environmental factors seem to play a role as a barrier or enabler to using the outdoor area, such as design of the outdoor area, staffing and resident safety, weather and seasons, design of the main building, and social activities (Sociaal en Cultureel Planbureau & Centraal Bureau voor de Statistiek, 2020; van den Berg et al., 2020).

Identifying characteristics that act as barriers or enablers to use the outdoor area is crucial to adequately reduce barriers and develop interventions to make going outside a normal part of daily nursing home practice for people living with dementia. To the authors’ knowledge, there is no data specifically for the population of people living with dementia regarding the frequency of going outside and which resident characteristics are independently associated with it. Therefore, the present study aims to explore the frequency of going outside and the characteristics that are associated with going outside for people living with dementia in nursing homes in the Netherlands and addressed the following research question: “How often do people living with dementia in nursing homes go outside and which resident characteristics are associated with the frequency of going outside?”

## Methods

### Data Source, Setting, and Participants

The present study was part of a survey that aimed to provide a national overview of the life situation, perceived quality of life and care for older nursing home residents in the Netherlands, carried out by Statistics Netherlands (CBS) in collaboration with The Netherlands Institute for Social Research (SCP) (Sociaal en Cultureel Planbureau & Centraal Bureau voor de Statistiek, 2020; Verbeek-Oudijk & Koper, 2019). The methods are fully described in the study protocol of the survey in DANS (Sociaal en Cultureel Planbureau & Centraal Bureau voor de Statistiek, 2020). In summary, to ensure a representative sample of residents living in nursing homes in the Netherlands, this survey used a two-phase stratified sampling method. Between January 2^nd^ and December 31^st^ 2019, data was collected on a population of 1837 residents of 55 years or older in 353 nursing homes (Sociaal en Cultureel Planbureau & Centraal Bureau voor de Statistiek, 2020). In the first phase, institutions were selected based on their size, with probabilities proportional to size. The selection was stratified by province to ensure representation from across the country. The selected institutions were then contacted by telephone for participation. Once an institution agreed to participate, a second-phase sample was drawn within that institution. A simple random sample of 12 residents was selected. The first 8 residents were included in the study sample and the remaining 4 residents served as reserves in case of eligibility issues. This design ensured that all residents in the target population had an equal chance of being included in the sample.

Participants were interviewed verbally, and if they were not capable of answering the questions themselves, (part of) the questions were submitted in writing to the care provider with primary responsibility for the participant, and the other part to a family member (Sociaal en Cultureel Planbureau & Centraal Bureau voor de Statistiek, 2020). Written informed consent was obtained from the participants or family members and caregivers who acted as a proxy for participants living with dementia.

The present study used a subsample of the original study. Only participants living with dementia for whom the question about frequency of going outside was answered were included (*N* = 693). In this subsample, all questions were answered by proxies (family members and caregivers).

### Outcome Measures

#### Frequency of Going Outside

The primary outcome measure was frequency of going outside and was operationalized in the question: “How often does the resident normally go outside, if the weather permits?” This question could be answered with “daily, weekly, monthly, or rarely or never.” The purpose of going outside did not matter, as long as the resident is going outside the nursing home building. They could, for example, take a walk around the building or just sit and take a nap in the nursing home garden. The participants could go outside alone, accompanied, or both.

#### Selection of Characteristics Associated with Frequency of Going Outside

Based on literature (Detweiler et al., 2009; Evans et al., 2019; Hendriks et al., 2016; Murphy et al., 2010; Nicholas et al., 2019; van den Berg et al., 2020; van der Velde-van Buuringen et al., 2020; Velde-van Buuringen et al., 2023) and clinical experience of the authors, potential correlates for the frequency of going outside were selected and categorized in sociodemographic characteristics, health and function-related characteristics, external factors, wishes and satisfaction, and quality of life.

#### Sociodemographic Characteristics

For this study, data were collected on age, gender, having a partner, level of urbanization, receiving visits, and receiving informal care.

#### Health and Function-Related Characteristics

Information on severity of physical impairment, need of walking aid, number of chronic diseases, pain medication, medication for psychological problems, pain, and sleeping problems was gathered. Also collected was information about how health in general hindered the frequency of going outside.

#### External Factors, Wishes, and Satisfaction

Data were collected on external factors such as a lack of company or transport, bad weather, or no available staff to assist the participant with going outside. Information on wanting to go outside more often as well as satisfaction with the outside area was also collected.

#### Quality of Life

Quality of life (QoL) was assessed with the QUALIDEM, which includes 40 items that apply to people living with mild to severe dementia in nine QoL domains (care relationships, positive affect, negative affect, restless tense behavior, positive self-image, social relations, social isolation, feeling at home, and having something to do) (Ettema et al., 2007). Two caregivers scored the items after an observation period of one week. The QUALIDEM has satisfactory reliability (rho ranging from .60 to .90) and validity (Cronbach’s alpha ranging from .59 to .89) (Bouman et al., 2011; Ettema et al., 2007).

### Statistical Analysis

Participants were stratified into an “often” and a “rarely or never” go outside group based on whether the participants go outside daily/weekly (often) or monthly/rarely or never (rarely or never). The Kruskal–Wallis test and the Pearson’s chi-square test were used to assess differences between resident characteristics. A *p*-value of ≤.05 was considered statistically significant.

All categorical factors were dichotomized by merging categories. In four categories (severity physical impairment, number of chronic diseases, pain, and sleeping problems), dummy variables were created.

For each characteristic, we performed a univariate logistic regression analysis with the frequency of going outside as the dependent variable. Subsequently, a stepwise backward procedure was used for the multivariate regression model, including variables with a *p*-value of ≤.30. This threshold was chosen to ensure that potential correlates for the frequency of going outside were not excluded prematurely. While a *p*-value of ≤.30 may include variables with weaker predictive value, it is allowed for a more comprehensive exploration of the data. This approach ensures that variables which might become significant in the presence of others are considered, particularly in analyses where the relationships between predictors may not be straightforward (Hosmer, 2013).

In the final multivariate regression model, only variables with a *p*-value of ≤.05, without multicollinearity, were accepted.

All analyses were performed with SPSS PASW Statistics, version 18.0.0, 2015 (SPSS Inc, IBM, Chicago, IL).

## Results

### Study Population

Table 1 presents the characteristics of the participants who go outside often versus rarely or never go outside. Most participants were female (75.2%), without a partner (76.6%) and had a median age of 86.0 years (IQR 81.0–91.0). Approximately two-thirds (66.5%) of the participants go outside often.

**Table 1.** Characteristics of the Study Population (*n* = 693).

Characteristics	Total Group	Frequency of Going Outside		*p*-Value^‡^
		Often	Rarely or Never	
Sociodemographic variables				
Frequency, *n (*%*)*	693 (100)	461 (66.5)	232 (33.5)	-
Age in years, median (*IQR*)	86.0 (81.0–91.0)	86.0 (80.0–90.8)	87.0 (82.0–91.0)	.012^†^^§^
Female, *n (*%*)*	521 (75.2)	346 (75.1)	175 (75.4)	.914^||^
Partner, *n (*%*)*	162 (23.4)	106 (23.0)	56 (24.1)	.737 ^||^
Urbanized, *n (*%*)*	364 (52.5)	228 (49.5)	136 (58.6)	.023^† ||^
Receiving minimum of daily/weekly visits^a^, *n (*%*)*	492 (71.8)	340 (74.6)	152 (66.4)	.025^† ||^
Receiving informal care^a^, *n (*%*)*	609 (88.3)	419 (91.3)	190 (82.3)	.001* ^||^
Health and function-related characteristics				
Severity physical impairment^a^, *n (*%*)*				<.001* ^||^
- None/light/moderate	75 (10.9)	70 (15.3)	5 (2.2)	
- Severe	299 (43.4)	210 (45.9)	89 (38.5)	
- Very severe	315 (45.7)	178 (38.9)	137 (59.3)	
Need of walking aid^a^, *n (*%*)*	420 (61.9)	277 (60.7)	143 (64.1)	.394 ^||^
Number of chronic diseases, *n (*%*)*				.173 ^||^
- 1	49 (7.1)	37 (8.0)	12 (5.2)	
- 2–4	402 (58.0)	272 (59.0)	130 (56.0)	
- ≥5	242 (34.9)	152 (33.0)	90 (38.8)	
Medication^b^, *n (*%*)*				
- Pain	315 (50.6)	188 (46.0)	127 (59.6)	.005* ^||^
- Psychological problems	218 (35.0)	140 (34.2)	78 (36.6)	.554 ^||^
Pain^a^, *n (*%*)*				.018^† ||^
- Rarely or never	281 (41.5)	204 (45.2)	77 (34.1)	
- Sometimes	220 (32.5)	140 (31.0)	80 (35.4)	
- Often	176 (26.0)	107 (23.7)	69 (30.5)	
Sleeping problems^c^, *n (*%*)*				.125 ^||^
- Rarely or never	290 (51.4)	200 (52.5)	90 (49.2)	
- Sometimes	157 (27.8)	111 (29.1)	46 (25.1)	
- Often	117 (20.7%)	70 (18.4)	47 (25.7)	
Frequency of going outside hindered by^e^, *n (*%*)*				
- Health	193 (60.9)	121 (57.9)	72 (66.7)	.129 ^||^
External factors, wishes and satisfaction				
Frequency of going outside hindered by^e^, *n (*%*)*				
- Lack of transportation	76 (24.0)	49 (23.4)	27 (25.0)	.759 ^||^
- Lack of company	82 (25.9)	62 (29.7)	20 (18.5)	.032^† ||^
- Other	66 (20.8)	38 (18.2)	28 (25.9)	.108 ^||^
Want to go outside more often^c^, *n (*%*)*	279 (54.9)	200 (57.0)	79 (50.3)	.163 ^||^
Satisfied with outside area^a^, *n (*%*)*	566 (82.9)	390 (85.3)	176 (77.9)	.015^† ||^
Quality of life				
Quality of life^c^, QUALIDEM median (*IQR*)				
- Care relationship	17 (13–20)	17 (14–20)	16 (13–20)	.182 ^§^
- Positive affect	15 (12–18)	16 (13–18)	14 (11–18)	.001* ^§^
- Negative affect	6 (5–6)	6 (5–6)	6 (5–6)	.303 ^§^
- Restless tense behavior	6 (3–8)	6 (3–8)	6 (4–8)	.539 ^§^
- Positive self-image	8 (6–9)	8 (6–9)	8 (6–9)	.361 ^§^
- Social relations	12 (9–15)	12 (9–15)	11 (8–13.25)	<.001* ^§^
- Social isolation	7 (6–9)	8 (5–9)	7 (6–9)	.402 ^§^
- Feeling at home	11 (9–12)	11 (9–12)	11 (10–12)	.055 ^§^
- Having something to do	2 (0–3)	2.5 (1–4)	2 (0–3)	<.001* ^§^

*p ≤ .01, ^†^*p* ≤ .05.

^‡^*p*-value between residents who go outside often versus rarely or never; ^§^Kruskal–Wallis test; ^||^ Pearson’s chi-square test.

IQR: interquartile range.

^a^= Missing (*n*) 3–20.

^b^= Missing (*n*) 20–100.

^c^= Missing (*n*) 100–210.

^d^= Missing (*n*) 210–300.

^e^= Missing (*n*) 300–376.

### Univariate Analyses

Participants who go outside often were slightly younger (86.0 years of age, IQR 80.0–90.8; vs. 87.0 years of age, IQR 82.0–91.0), lived in an urbanized environment less often (49.5%; vs. 58.6%), received informal care more often (91.3%; vs. 82.3%), and received a minimum of daily or weekly visits more often (74.6%; vs. 66.4%) than participants who rarely or never go outside (see Table 2). Also, they used pain medication less often (46.0%; vs. 59.6%), were prevented from going outside more frequently due to a lack of company more often (29.7%; vs. 18.5%), and were satisfied with the outside environment more often (85.3%; vs. 77.9%), compared to participants who rarely or never go outside. They had higher scores on the QUALIDEM domains of positive affect (16, IQR 13–18; vs. 14, IQR 11–18), social relations (12, IQR 9–15; vs. 11, IQR 8–13.25), and having something to do (2.5, IQR 1–4; vs. 2, IQR 0–3). No significant differences were found for the other outcomes.

**Table 2.** Univariate Logistic Regression for Each Potentially Correlated Characteristic to the Frequency of Going Outside.

	OR	95% CI	*p*-Value**
Sociodemographic variables			
Age (continuous)	0.97	0.95–0.99	0.010*
Gender (female vs. male)	0.98	0.68–1.41	0.914
Partner (yes vs. no)	0.94	0.65–1.36	0.737
Urbanized (somewhat to not urbanized vs. urbanized)	1.45	1.05–1.99	0.023^†^
Receiving visits (daily/weekly vs. less than daily/weekly)	1.49	1.05–2.10	0.025^†^
Receiving informal care (yes vs. no)	2.26	1.42–3.61	0.001*
Health and function-related characteristics			
Severity physical impairment			
- (Severe vs. none/light/moderate)	0.17	0.07–0.43	<.001*
- (Very severe vs. none/light/moderate)	0.09	0.04–0.24	<.001*
Need of walking aid (yes vs. no)	0.87	0.62–1.12	0.395
Number of chronic diseases			
- (2–4 vs. 1)	0.68	0.34–1.35	0.266^||^
- (≥5 vs. 1)	0.55	0.27–1.11	0.093^‡^
Medication for pain (yes vs. no)	0.56	0.39–0.80	0.001*
Medication for psychological problems (yes vs. no)	0.87	0.61–1.24	0.432
Pain			
- (Sometimes vs. rarely or never)	0.66	0.45–0.97	0.032^†^
- (Often vs. rarely or never)	0.59	0.39–0.87	0.009*
Sleeping problems			
- (Sometimes vs. rarely or never)	1.09	0.71–1.66	0.704
- (Often vs. rarely or never)	0.67	0.43–1.05	0.078^‡^
Frequency of going outside hindered by health (yes vs. no)	0.69	0.42–1.12	0.130^§^
External factors, wishes and satisfaction			
Frequency of going outside hindered by lack of transportation (yes vs. no)	0.92	0.54–1.58	0.759
Frequency of going outside hindered by lack of company (yes vs. no)	1.86	1.05–3.28	0.033^†^
Frequency of going outside hindered by other reason (yes vs. no)	0.64	0.36–1.11	0.109^§^
Want to go outside more often (yes vs. no)	1.31	0.90–1.91	0.164^§^
Satisfied with outside area (yes vs. no)	1.65	1.10–2.49	0.015^†^
Quality of life (QUALIDEM)			
Care relationship (continuous)	1.03	0.99–1.08	0.160^§^
Positive affect (continuous)	1.10	1.05–1.16	<.001*
Negative affect (continuous)	1.08	0.93–1.27	0.316
Restless tense behavior (continuous)	0.98	0.92–1.04	0.535
Positive self-image (continuous)	1.05	0.96–1.15	0.308
Social relations (continuous)	1.10	1.05–1.15	<.001*
Social isolation (continuous)	1.03	0.96–1.12	0.399
Feeling at home (continuous)	0.90	0.83–0.98	0.016^†^
Having something to do (continuous)	1.25	1.12–1.39	<.001*

*p ≤ .01, ^†^*p* ≤ .05, ^‡^*p* ≤ .10, ^§^*p* ≤ .20, ^||^*p* ≤ .30.

**p-value between residents who go outside often versus rarely or never.

OR: odds ratio; CI: confidence interval.

### Multivariate Analyses

In the final multivariate model, five variables remained that are independently associated with the frequency of going outside (see Table 3; *n* = 388). Participants who receive visits daily or weekly were 1.68 times more likely to go outside often as compared to those receiving visits less frequently (odds ratio (OR) 1.68; 95% confidence interval (CI) 1.02–2.75). Participants with severe physical impairment were 0.17 times less likely (OR 0.17; 95% CI 0.04–0.73), and participants with very severe physical impairment were 0.11 times less likely (OR 0.11; 95% CI 0.03–0.49), to go outside often than those with no, mild, or moderate physical impairment. Participants taking medication for pain were 0.61 times less likely (OR 0.61, 95% CI 0.38–0.98) to go outside often compared to those not taking medication. Positive affect was positively associated with going outside often (OR 1.10; 95% CI 1.03–1.17), suggesting that when the score of positive affect increases by one point, participants were 1.10 times more likely to go outside often. Feeling at home was negatively associated with going outside often (OR 0.86; 95% CI 0.76–0.97), suggesting that when the score of feeling at home increases by one point, participants were 0.86 times less likely to go outside often.

**Table 3.** Final Multivariate Model for the Frequency of Going Outside (*n* = 388).

Variable	B	OR	95% CI	*p*-Value*
Receiving visits (daily/weekly vs. less than daily/weekly)	0.52	1.68	1.02–2.75	0.040
Severity physical impairment				0.007
- (Severe vs. none/light/moderate)	−1.80	0.17	0.04–0.73	0.017
- (Very severe vs. none/light/moderate)	−2.21	0.11	0.03–0.49	0.004
Medication for pain (yes vs. no)	−0.50	0.61	0.38–0.98	0.039
QoL positive affect (continuous)	0.09	1.10	1.03–1.17	0.005
QoL feeling at home (continuous)	−0.15	0.86	0.76–0.97	0.011

*p-value between residents who go outside often versus rarely or never.

OR: odds ratio; CI: confidence interval.

The Nagelkerke R square was 0.172, that is, the model explains 17.2% of the variability in the frequency of going outside. Further analyses showed that the variable severity of physical impairment explained the largest portion of the variability in the frequency of going outside.

## Discussion

The present study aimed to explore the frequency of going outside and the characteristics that are associated with going outside for people living with dementia in nursing homes. The results show that approximately two-thirds of the participants go outside often, and one-third go outside rarely or never. Five factors were independently associated with the frequency of going outside. The findings revealed that participants who go outside often receive daily or weekly visits, have a higher positive affect, have less severe physical impairment, do not take medication for pain, and feel less at home.

This is one of the first studies to examine possible characteristics of people living with dementia in nursing homes that are associated with the frequency of going outside. Many previous studies focused on the effects of going outside, not on characteristics. Despite this difference, the findings of our study appear to be in line with the key themes of the results of these previous studies (Evans et al., 2019; Hendriks et al., 2016; van der Velde-van Buuringen et al., 2020). For example, one study showed that people living with dementia in nursing homes experience pleasure, relaxation, feeling fit, enjoying the beauty of nature, feeling free, the social aspect of nature, feeling useful, and memories, as being important for their QoL when outside (Hendriks et al., 2016). In another study, staff members highlighted numerous ways in which nature-based activities positively impacted the QoL of people living with dementia in nursing homes. These included high levels of engagement, a sense of freedom, creativity, increased social interaction, inter-generational contact with families, and the calming effect of contact with animals (Evans et al., 2019). In addition, a feasibility study found that people living with dementia showed increased positive affect and decreased social isolation on the QUALIDEM during an intervention period of going outside (van der Velde-van Buuringen et al., 2020).

Given that severity of physical impairment explained the largest proportion of the variability in the frequency of going outside, interventions should start with focusing on addressing this important factor. The systematic review of van den Berg et al. (2020) revealed the importance of a well-designed physical environment to enable and support garden use by residents with physical impairments. Their results showed that the design of the outside area was the most frequently mentioned main barrier or enabler across all included studies. Multiple, easy-to use-access points, safety elements such as glare-free and slip-resistant pathways with handrails, and appropriate lighting are necessary to help residents with physical impairments overcome mobility problems and gain access to the outside area (Brawley, 2007; Kearney & Winterbottom, 2006; van den Berg et al., 2020). Including elements that contribute to a sense of familiarity could also help the outside area contribute to a sense of home (Pollock, 2012; van den Berg et al., 2020). In addition, interventions that focus on stimulating and facilitating residents to engage in more social activities could improve the social system around the resident and positively influence the frequency of visits from family and friends. A well-designed outside area with organized programmed activities can facilitate this (Dyer et al., 2021; van den Berg et al., 2020). Combining these elements by creating an easy-to-access, safe, familiar, green, and natural outside area, with organized programmed activities, may generate a feeling of satisfaction with the outside area.

This study has some considerable strengths, and one being that it used a subsample of a national survey. The two-phase stratified sampling method to collect this survey data minimizes biases and ensures high quality of the collected information. Also, the large sample, with a population of 1837 residents in 353 nursing homes, is a strength. Therefore, findings of this study may be generalizable to other nursing homes in the Netherlands that were not included in this sample. However, variations in operational standards, cultural contexts, and climate may influence the applicability of these findings across different settings. To date, limited research has addressed generalizability in this field. Goto et al. (2018) examined the effects of exposure to a Japanese garden across different locations, countries, cultures, and ethnic groups, demonstrating that the positive effects of garden observation were consistent across these varying environments. While these findings are encouraging, more research is necessary to fully understand the applicability of the results from the current study in other settings.

There are also some limitations. The study used a cross-sectional design, which limits the possibility to establish causal relationships between the identified characteristics and the frequency of going outside. It is therefore impossible to say whether the significant characteristics found are traits of the people living with dementia in nursing homes themselves that cause them to go outside more often, or that these factors are a result of going outside more often, or that an unknown factor influences both. Future longitudinal studies should provide a better understanding of these temporal associations between these characteristics. In addition, a Nagelkerke R square of 0.172 that explains 17.2% of the variability in the frequency of going outside still leaves 82.8% of the variability unexplained. Also, the study relied solely on proxy responses from family members and caregivers, which may introduce response biases (Macháčová et al., 2018; Neumann et al., 2000). These biases can influence the results and affect the validity of the findings, as proxies may have different perceptions or levels of awareness regarding the residents’ true preferences and experiences (Howland et al., 2017; Ruggero et al., 2023). Future research could focus on mitigation strategies, such as using observational methods, to provide more accurate information. Furthermore, “going outside” was not explicitly defined in the questionnaire. The question asked proxies to indicate how often the resident typically goes outside when the weather permits. The lack of a precise definition may introduce some ambiguity into the interpretation of responses. Future studies could consider providing clearer definitions to gain a more accurate understanding of residents’ outdoor activities. Last, by stratifying the participants into an “often” go outside and a “rarely or never” go outside group, the authors chose to qualify going outside weekly as “often.” This stratification may have influenced the results.

## Conclusions and Implications

This study indicates that two in three people living with dementia in nursing homes go outside often. Receiving visits, pain medication, and the level of physical impairments, positive affect, and feeling at home were independently associated with the frequency of going outside. These findings are the first step in developing effective interventions that will contribute to people living with dementia going outside more often.
